# Mortality in Biopsy-Confirmed Nonalcoholic Fatty Liver Disease: Results From A Nationwide Cohort

**DOI:** 10.1136/gutjnl-2020-322786

**Published:** 2020-10-09

**Authors:** Tracey G. Simon, Bjorn Roelstraete, Hamed Khalili, Hannes Hagström, Jonas F. Ludvigsson

**Affiliations:** 1 Division of Gastroenterology and Hepatology, Massachusetts General Hospital, Boston, MA, USA; 2 Harvard Medical School, Boston, MA, USA; 3 Clinical and Translational Epidemiology Unit (CTEU), Massachusetts General Hospital, Boston, MA, USA; 4 Department of Medical Epidemiology and Biostatistics, Karolinska Institutet, Stockholm, Sweden; 5 Division of Hepatology, Department of Upper GI Diseases, Karolinska University Hospital, Stockholm, Sweden; 6 Clinical Epidemiology Unit, Department of Medicine Solna, Karolinska Institutet, Stockholm, Sweden; 7 Department of Pediatrics, Orebro University Hospital, Orebro, Sweden.; 8 Division of Epidemiology and Public Health, School of Medicine, University of Nottingham, UK.; 9 Department of Medicine, Columbia University College of Physicians and Surgeons, New York, New York, USA

**Keywords:** epidemiology, fatty liver, fibrosis, hepatocellular carcinoma, liver cirrhosis

## Abstract

**Objective:**

Population-based data are lacking regarding the risk of overall and cause-specific mortality across the complete histological spectrum of nonalcoholic fatty liver disease (NAFLD).

**Design:**

This nationwide, matched cohort study included all individuals in Sweden with biopsy-confirmed NAFLD (1966–2017; n=10,568). NAFLD was confirmed histologically from all liver biopsies submitted to Sweden’s 28 pathology departments, after excluding other etiologies of liver disease, and further categorized as, simple steatosis, non-fibrotic steatohepatitis (NASH), non-cirrhotic fibrosis and cirrhosis. NAFLD cases were matched to ≤5 general population comparators by age, sex, calendar year and county (n=49,925). Using Cox regression, we estimated multivariable-adjusted hazard ratios (aHRs) and 95%CIs.

**Results:**

Over a median of 14.2 years, 4,338 NAFLD patients died. Compared to controls, NAFLD patients had significantly increased overall mortality (16.9 vs. 28.6/1000 person-years [PY]; difference=11.7/1000PY; aHR=1.93, 95%CI=1.86–2.00). Compared to controls, significant excess mortality risk was observed with simple steatosis (8.3/1000PY, aHR=1.71, 95%CI=1.64–1.79), non-fibrotic NASH (13.4/1000PY, aHR=2.14, 95%CI=1.93–2.38), non-cirrhotic fibrosis (18.4/1000PY, aHR=2.44, 95%CI=2.22–2.69) and cirrhosis (53.6/1000PY, aHR=3.79, 95%CI=3.34–4.30)(P_trend_<0.01). This dose-dependent gradient was similar when simple steatosis was the reference (P_trend_<0.01). The excess mortality associated with NAFLD was primarily from extra-hepatic cancer (4.5/1000PY; aHR=2.16, 95%CI=2.03–2.30), followed by cirrhosis (2.7/1000PY; aHR=18.15, 95%CI=14.78–22.30), cardiovascular disease (1.4/1000PY; aHR=1.35, 95%CI=1.26–1.44) and hepatocellular carcinoma (HCC)(1.2/1000PY; aHR=11.12, 95%CI=8.65–14.30).

**Conclusions:**

All NAFLD histological stages were associated with significantly increased overall mortality, and this risk increased progressively with worsening NAFLD histology. Most of this excess mortality was from extra-hepatic cancer and cirrhosis, while in contrast, the contributions of cardiovascular disease and HCC were modest.

## Introduction

Non-alcoholic fatty liver disease (NAFLD) represents the most common cause of chronic liver disease in Western countries, affecting nearly 25% of U.S. and European adults^1,2^. Nearly one-third of patients with NAFLD develop progressive steatohepatitis (NASH) and fibrosis, which can lead to cirrhosis, decompensated liver disease, and death^3–5^. Small clinical studies have demonstrated that among patients with NAFLD, advanced liver fibrosis, rather than inflammatory NASH, is the most important histological predictor of survival^4–8^. Accordingly, current guidelines recommend that patients with NAFLD undergo risk stratification according to the presence or absence of significant fibrosis^7^. However, robust, population-level data to support this strategy are lacking. Published evidence linking NAFLD histology to survival derives exclusively from studies with small, selected populations of less than 650 subjects and which recorded relatively few deaths, resulting in imprecise risk estimates and limited ability to comprehensively assess mortality^4,5,8–12^. Thus, the precise impact of NAFLD histology on the long-term risk of overall and cause-specific mortality is still undefined. Given the growing burden of NAFLD, leveraging population-level data to quantify the magnitude of these risks is important for developing more effective strategies for prevention, surveillance and intervention^13^.

Thus, we evaluated the risks of overall and cause-specific mortality according to the presence and histological severity of NAFLD, in a population-based cohort comprised of all adults in Sweden with biopsy-confirmed NAFLD. With complete, nationwide histopathology data and over 30 years of long-term follow-up, this cohort permits a more comprehensive assessment of mortality risk across the full histological spectrum of NAFLD.

## Methods

### Study Population & Exposure

We conducted a population-based, matched cohort study using the ESPRESSO (Epidemiology Strengthened by Histopathology Reports in Sweden) cohort. ESPRESSO includes prospectively-recorded liver histopathology data from all 28 Swedish pathology departments (1966–2017), and therefore is complete for the entire country of Sweden^14^. Each report includes a unique personal identity number (PIN), biopsy date, and as well as topography within the liver, and morphology. We then linked ESPRESSO to validated registers containing prospectively-recorded data regarding demographics, comorbidities, prescribed medications and death. ESPRESSO was approved by the Stockholm Ethics Board on August 27, 2014 (No.2014/1287-31/4). Informed consent was waived as the study was register-based^15^.

We identified all liver biopsy specimens from adults aged ≥18 years, submitted between 1966–2017, with topography codes corresponding to the liver, and Systematized Nomenclature of Medicine (SNOMED) codes corresponding to steatosis, without any other recorded etiology of liver disease (eMethods). Using a validated algorithm, we excluded anyone with another etiology of liver disease, prior history of alcohol abuse/misuse, liver transplantation or emigration from Sweden before the index date, or with <180 days of follow-up (Figure S1). We further categorized NAFLD patients into 4 histological groups (i.e. simple steatosis; NASH without fibrosis; non-cirrhotic fibrosis; cirrhosis)(eMethods).

### Validation

We completed a validation study of 149 randomly-selected adults meeting criteria for biopsy-confirmed NAFLD. A physician (JFL) confirmed 137/149 to be NAFLD by reviewing free text from the pathologist (positive predictive value [PPV] of 92%). Additionally, we evaluated 119 different, randomly-selected adults, and obtained PPVs of 90% (27/30) for simple steatosis, 87% (27/31) for NASH without fibrosis, 93% (28/30) for non-cirrhotic fibrosis and 97% (27/28) for cirrhosis.

### Comparators

Each NAFLD patient was matched to up to 5 general population comparators without recorded NAFLD, according to age, sex, calendar year and county. Comparators were derived from the Total Population Register^16^, and identical exclusion criteria were applied (Figure S1).

### Outcomes & Covariates

All-cause mortality was ascertained from the Total Population Register, which prospectively records 93% of all deaths within 10 days, and the remaining 7% within 30 days^17^. Specific causes of death were retrieved from the Cause of Death Register^17^, and categorized as: hepatocellular carcinoma (HCC) mortality, extra-hepatic (i.e. non-HCC) cancer mortality, cirrhosis mortality (excluding HCC), cardiovascular mortality, and other causes of death (defined in eMethods and Table S2).

Table S2 and the eMethods contain detailed data regarding demographic, clinical and medication covariates. We ascertained age, sex, date of birth and emigration from the Total Population Register^16^. Education level was obtained from the LISA database^18^. Clinical comorbidities were collected from the Patient Register, which prospectively records all data from hospitalizations (including surgeries), discharge diagnoses (1964- ) and specialty outpatient care (2001- ), and is well-validated, with PPVs for clinical diagnoses that are consistently 85–95%^19^. The Prescribed Drug Register has prospectively recorded all prescriptions dispensed from Swedish pharmacies since 2005, and is well-validated and virtually complete^20^, permitting accurate and comprehensive ascertainment of relevant medications, including statins, low-dose aspirin (<163mg), antidiabetic and anti-hypertensive agents^20^.

### Statistical Analysis:

Our primary analyses evaluated all-cause and cause-specific mortality in patients with NAFLD compared to matched population controls, and according to NAFLD histological severity. Follow-up began ≥180 days after the index date, and continued to the first recorded date of death, emigration, or end of follow-up (December 31, 2017; cause-specific mortality, December 31, 2016). Population comparators who subsequently developed NAFLD were censored at that diagnosis date, and subsequently contributed person-time in the NAFLD group.

We constructed Kaplan-Meier curves to calculate incidence rates and absolute rate differences with 95% confidence intervals (CIs). We also calculated 20-year absolute risks and risk differences, with 95%CIs approximated by the normal distribution. Using Cox proportional hazard models, we estimated multivariable adjusted hazard ratios (aHRs), accounting for *a priori*-defined covariates (i.e. age, sex, county, calendar year, education level, cardiovascular disease and the metabolic syndrome [as a 5-level variable: 1-point for diabetes, obesity, hypertension and/or dyslipidemia])(Table S2). The proportional hazards assumption was assessed by examining the association between Schoenfeld residuals and time.

To assess specific underlying causes of mortality, we constructed cause-specific regression models. Further, because cause-specific mortality may be overestimated in the setting of competing events^21^, we repeated this analysis after accounting for other causes of death as competing risks. In stratified models, we examined the associations between NAFLD and both all-cause and cause-specific mortality according to known and putative risk factors for mortality, and we tested the significance of effect modification using the log likelihood ratio test.

To further characterize the potential gradient of mortality risk associated with progressive NAFLD histological severity, and to minimize potential bias related to the original indication for liver biopsy, we restricted the cohort to patients with histologically-defined NAFLD, with simple steatosis as the comparator. Additionally, because patients with advanced fibrosis were older than those with simple steatosis, we repeated this analysis after re-matching patients with simple steatosis 1:1 to individuals in each of the other NAFLD groups, by age(+/−2 years), sex, calendar year and county.

We conducted numerous sensitivity analyses to test the robustness of our results. First, we repeated our primary analyses after re-matching NAFLD patients to unaffected full siblings without NAFLD^16^, to address potential confounding related to shared genetic or early environmental factors. Second, because a widely-used NAFLD histological scoring system was published in 2005^22^, the year that medication data became available in Sweden, we restricted the cohort to index date ≥January 1, 2006, and we adjusted for time-varying use of aspirin, statin and anti-diabetic medications in our multivariable models. Third, to further address potential residual confounding, we constructed models additionally accounting for a modified Charlson Comorbidity Index (eMethods), and also for incident diagnoses of alcohol abuse/misuse during follow-up (Table S1). Fourth, we censored anyone diagnosed with cancer within ≤180 days of follow-up, or anyone who died within <2 years. Finally, to further address potential residual confounding, we tested the sensitivity of our data to an unmeasured confounder^23^.

Statistical analyses were conducted using R software (version 3.6.1, R Foundation for Statistical Computing, Vienna, Austria; and survival package version 2.44 (Therneau, 2015, https://CRAN.R-project.org/package=survival)). A two-sided P<0.05 was considered statistically significant.

## Patient and Public Involvement

No patients were involved in setting the research question or the outcome measures. However, patients were involved in the establishment of the overall ESPRESSO cohort, which formed the foundation of this work. No patients were asked to advise on interpretation or writing up of results. The results of this research will be disseminated to patients by press release.

## Results

Among 10,568 adults with histologically-confirmed NAFLD, 7,105 (67.2%) had simple steatosis, 1,218 (11.5%) had NASH without fibrosis, 1,658 (15.7%) had non-cirrhotic fibrosis, and 587 (5.6%) had cirrhosis (Table 1). Among NAFLD patients, the average age at index biopsy was 52 years, and 44.8% were female. Compared to population comparators, NAFLD patients were more likely to have cardiovascular disease, diabetes, hypertension and dyslipidemia (Table 1). Median follow-up was 14.2 years among NAFLD patients, and 16.8 years among population comparators.

### All-Cause Mortality

**Figure 1.**
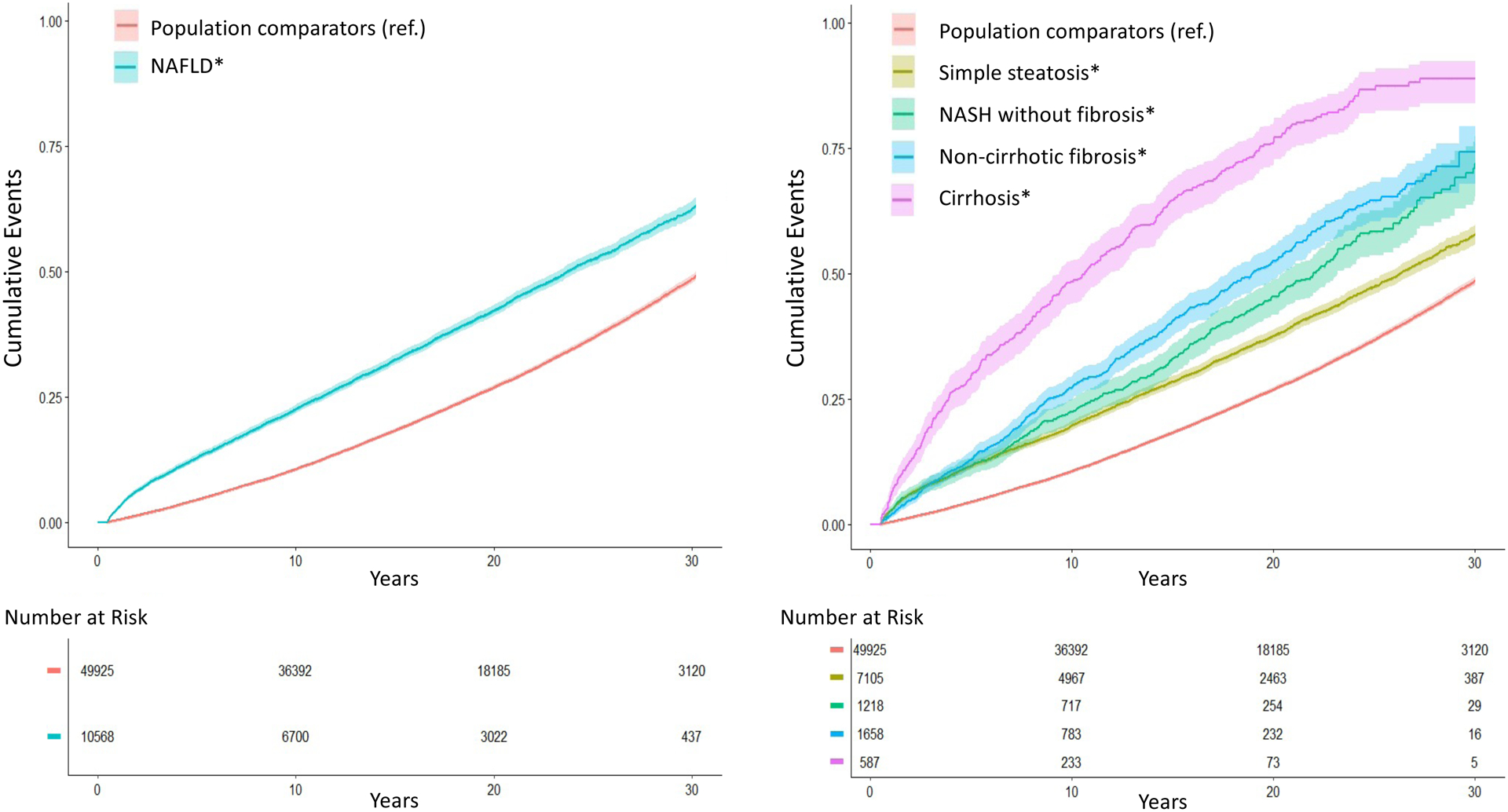
Cumulative Incidence of All-Cause Mortality According to the Presence and Histologic Severity* of NAFLD Abbreviations: NAFLD, nonalcoholic fatty liver disease; NASH, nonalcoholic steatohepatitis; ref., reference group *NAFLD histologic severity was defined in 4 categories, as simple steatosis, NASH without fibrosis, non-cirrhotic fibrosis and cirrhosis (for details, see the eMethods section)

Overall, we documented 4,338 deaths among NAFLD patients (28.6/1000 person-years [PY]), and 13,911 deaths among comparators (16.9/1000PY) yielding an absolute rate difference of 11.7/1000PY, and a 20-year absolute risk difference of 15.3% (95%CI=13.3–17.3)(Table 2). After multivariable adjustment, NAFLD patients had a 1.93-fold higher risk of overall mortality, compared to population comparators (95%CI=1.86–2.00)(Figure 1; Table 2). The significant, positive association between NAFLD and increased risk of overall mortality was similar among women and men, and in patients with and without cardiovascular disease, diabetes, dyslipidemia, hypertension or the metabolic syndrome (all P_heterogeneity_>0.05) (Figure S2). Hazard estimates for overall mortality were higher among patients diagnosed <60 years (vs ≥60 years), and those who died within the first 2 years of follow-up.

Mortality risk increased with worsening NAFLD severity (P_trend_<0.01)(Figure 1; Table 2). Compared to population controls, the absolute rates and corresponding aHRs for overall mortality were significantly elevated in all NAFLD patients, including those with simple steatosis (8.3/1000PY; aHR=1.71, 95%CI=1.64–1.79), NASH without fibrosis (13.4/1000PY; aHR=2.14, 95%CI=1.93–2.38), non-cirrhotic fibrosis (18.4/1000PY; aHR=2.44, 95%CI=2.22–2.69) and cirrhosis (53.6/1000PY; aHR=3.79, 95%CI=3.34–4.30). After 20 years, this corresponded to an absolute excess risk of overall mortality of 10.7% with simple steatosis, 18.5% with NASH without fibrosis, 25.6% with non-cirrhotic fibrosis, and 49.4% with cirrhosis, compared to population controls. These findings were similar in men and women, and in those with and without cardiovascular disease, diabetes, hypertension, dyslipidemia and the metabolic syndrome (all P_heterogeneity_>0.05; not shown).

### Cause-Specific Mortality

In both NAFLD patients and population controls, extra-hepatic cancer and cardiovascular disease represented the two most common causes of death. Compared to controls, NAFLD patients had significantly higher rates of cause-specific mortality due to extra-hepatic cancer (4.8 vs. 9.3/1000PY; aHR=2.16, 95%CI=2.03–2.30), followed by cirrhosis (0.2 vs. 2.8/1000PY; aHR=18.15, 95%CI=14.78–22.30), cardiovascular disease (6.9 vs. 8.3/1000PY; aHR=1.35, 95%CI=1.26–1.44) and HCC (0.1 vs. 1.3/1000PY; aHR=11.12, 95%CI=8.65–14.30)(Table 3). Deaths from other causes were also more common among patients with NAFLD.

We also evaluated cause-specific mortality according to NAFLD histological categories. Compared to population comparators, mortality rates from extra-hepatic cancer, cirrhosis, cardiovascular disease and HCC were modestly but significantly elevated in simple steatosis (absolute rate differences, 4.4, 1.2, 0.7 and 0.7/1000PY, respectively), and these rates increased progressively in NASH without fibrosis (3.9, 3.0, 2.7 and 1.3/1000PY, respectively), non-cirrhotic fibrosis (4.5, 5.5, 1.8 and 2.5/1000PY, respectively) and cirrhosis (6.5, 22.3, 8.2 and 5.5/1000PY, respectively). After accounting for potential competing events (i.e. other causes of death)^21^, we observed similar, dose-dependent gradients of increasing risk of extra-hepatic cancer-, cirrhosis- and HCC-related mortality, with worsening NAFLD histological severity, consistent with our primary analysis (all P_trend_<0.01)(Table S3). In contrast, after accounting for competing risks, NAFLD was no longer significantly associated with significant excess risk of cardiovascular mortality (aHR=0.98, 95%CI=0.92–1.04) nor was a dose-response relationship observed (P_trend_=0.75).

### NAFLD-Only Subgroup

After restricting the population to patients with biopsy-confirmed NAFLD, and using simple steatosis as the comparator, we observed a similar, dose-dependent relationship between worsening NAFLD histological severity and increased overall mortality (P_trend_<0.01; Table 4). Compared to simple steatosis, the aHRs with NASH without fibrosis, non-cirrhotic fibrosis and cirrhosis were, 1.14 (95%CI=1.03–1.26), 1.26 (95%CI=1.15–1.38), and 1.95 (95%CI=1.75–2.18), respectively.

We also assessed between-group differences in the absolute risk of overall mortality among patients with non-cirrhotic fibrosis, compared to those with NASH without fibrosis (Figure 1, **panel B**). At 10 years, the cumulative incidence of all-cause mortality was significantly higher among patients with non-cirrhotic fibrosis (27.2 percentage points [95%CI=25.6–28.9]) compared to NASH without fibrosis (22.5 percentage points [95%CI=20.8–24.1]; P_difference_=0.041). However, at 20 years, this difference was no longer statistically significant (20-year cumulative incidence in patients with non-cirrhotic fibrosis vs. NASH without fibrosis, 52.4 percentage points [95%CI=48.8–56.0] vs. 45.4 percentage points [95%CI=42.1–48.7]; P_difference_=0.15).

We also evaluated cause-specific mortality according to NAFLD severity, within this NAFLD-only subgroup. Compared to patients with simple steatosis, the 20-year absolute excess risks of liver-, cardiovascular- and HCC-specific mortality were significantly higher in patients with NASH without fibrosis (3.3, 4.4 and 1.7%, respectively), non-cirrhotic fibrosis (6.8, 4.9 and 4.0%, respectively) and cirrhosis (30.4, 16.0 and 11.1%, respectively); in contrast, no significant between-group differences were found for cancer-specific mortality (Table S4).

### Sensitivity analyses

Our findings were robust across all sensitivity analyses, including: (1) after matching NAFLD patients to full-sibling comparators (Tables S5); (2) after restricting the index date to ≥January 1, 2006, and further adjusting for time-varying medications (Table S6); (3) after constructing multivariable models additionally accounting for the modified Charlson Comorbidity Index (Table S7A**-**B), or incident alcohol abuse/misuse (aHR_mortality_ for NAFLD=1.85, 95%CI=1.78–1.91); and (4) after excluding anyone diagnosed with cancer within ≤180 days (n=6,258 excluded; aHR_mortality_=1.71, 95%CI=1.64–1.78). To further address potential reverse causation, and to account for the elevated HRs observed in persons with very short follow-up time, we also excluded anyone who died within <2 years of follow-up (n=1,342 excluded), and our results were similar (aHR_mortality_=1.76, 95%CI=1.69–1.83). Finally, we observed that an unmeasured confounder would have to be both very strongly associated with mortality and highly imbalanced (i.e. aHR<0.1 or ≥4.5, with >50% difference in prevalence), to fully attenuate our results (Table S8).

## Discussion

In this population-based cohort of 10,568 adults with biopsy-confirmed NAFLD and 49,925 matched general population comparators, NAFLD was associated with a 93% higher relative risk of overall mortality, and a 20-year absolute excess risk of 15.3%. Significantly elevated risk of overall mortality was apparent at all stages of NAFLD, and this risk increased in a dose-dependent manner with worsening histological severity. Specifically, 20-year absolute excess risk of mortality was 10.7% higher with simple steatosis, 18.5% higher with NASH without fibrosis, 25.6% higher with non-cirrhotic fibrosis, and 49.4% higher with cirrhosis, compared to the general population. This excess risk was due primarily to increased cancer- and cirrhosis-specific mortality, while the contributions of cardiovascular disease- and HCC-specific mortality were relatively modest.

Although previous studies have linked NAFLD fibrosis to increased risk of mortality^4,5,8–12^, those prior studies have been limited by small sample sizes, with few recorded deaths in each histological group, which yield imprecise risk estimates and poor generalizability^8–12^. For example, in one of the largest published studies, 619 patients with biopsy-confirmed NAFLD were followed for a median of 12.6 years, and liver transplant-free survival did not differ significantly between patients with simple steatosis and those with non-fibrotic NASH (P=0.238)^8^. However, that analysis included only 12 deaths in the non-fibrotic NASH group. In contrast, the current study leveraged a complete, nationwide population of all adults in Sweden with histologically-defined NAFLD, and included longer follow-up time and more recorded deaths (4,338) than all prior NAFLD histology cohorts, combined^4,5^.

Currently, it is widely held that among patients with NAFLD, liver fibrosis is the only significant histological predictor of survival^4,5,8–12^; however, robust population-level evidence to support this hypothesis is lacking^13^. Our data confirm this association in a nationwide, unselected population, and the significant, dose-response relationships that we observed across histological groups lend further support to a causal relationship. Furthermore, our large sample size permitted us to detect important differences in mortality rates between groups of patients with earlier stages of NAFLD, which was not possible in previous, smaller histology cohorts. Specifically, compared to patients with simple steatosis, those with non-fibrotic NASH had an excess mortality rate of 5.1 per 1000 person-years. While that figure might seem modest, over 20 years it translates to 1 additional death for every 10 patients diagnosed with non-fibrotic NASH. Thus, our findings suggest the need for more refined algorithms for risk stratification, surveillance and monitoring, for patients with early-stage NAFLD^7^.

It has been established that liver-related mortality increases progressively with worsening NAFLD fibrosis^4,5^. However, much less is known about the relationship between NAFLD histology and other specific causes of death. We observed that the increased mortality associated with NAFLD was driven primarily by excess risk of cancer- and cirrhosis-specific mortality, together with a small, albeit significant, excess risk of HCC-specific mortality. In contrast, the absolute excess risk of cardiovascular-specific mortality was modest, and it was no longer significant after accounting for competing events. Together, these data are consistent with recent studies highlighting the growing importance of fatal cancers and cirrhosis, as complications of NAFLD^4,11,24,25^, and which suggest that the relationship between NAFLD and cardiovascular mortality might be less important than previously suggested^24,26–31^. Indeed, while substantial evidence links NAFLD to an increased risk of non-fatal cardiovascular events^32^, whether NAFLD contributes to excess cardiovascular mortality remains controversial^33^. To date, two large meta-analyses have failed to demonstrate a significant association between NAFLD and cardiovascular mortality risk^29,34^. Although a third meta-analysis found that NAFLD was significantly associated with an increased risk of both fatal and non-fatal cardiovascular events, that relationship was no longer statistically significant when analyses focused specifically on cardiovascular mortality^35^. Thus, while it remains important to carefully assess cardiovascular disease risk in patients with NAFLD^7^, our data lend strong support to the development of public health efforts designed to prevent cancer and cirrhosis, for this growing patient population.

We considered whether the relationship between NAFLD and premature death merely reflected an association with the components of the metabolic syndrome. Consistent with other administrative datasets, the recorded prevalences of hypertension and obesity were low, which could lead to unmeasured confounding. Nevertheless, our findings remained similar in patients with and without these diagnoses, when compared to controls with the same comorbidities. Moreover, robust evidence demonstrates that hypertension, obesity and the metabolic syndrome contribute only modestly to excess mortality risk (adjusted HRs for hypertension, 1.09–1.37;^36–38^ for overweight/obesity, 0.94–1.18;^39^ and for the full metabolic syndrome, 1.58)^40^. Finally, our sensitivity analysis demonstrated that our results are robust to unmeasured confounding; specifically, a confounder would need to have both an adjusted HR≥4.5 for overall mortality and a >50% difference in prevalence between groups to attenuate our results. Thus, the excess mortality risk observed with NAFLD appears to far exceed that which could be explained by the metabolic syndrome, alone.

This study benefits from a nationwide, unselected population with complete and prospectively-recorded histopathological data for the entire country of Sweden. We used strict and validated definitions of both NAFLD and confounding variables, in registers with near-complete follow-up for the entire Swedish population^16^. Our large sample size and long follow-up permitted calculation of more precise, population-level risk estimates across NAFLD histological categories, while minimizing the inherent limitations of previous, smaller studies. Conducting analyses exclusively in patients with histologically-defined NAFLD further reduced potential exposure misclassification or bias related to the indication for biopsy. Using cause-specific hazards models allowed for more comprehensive analyses of underlying causes of mortality. We also applied numerous analytical techniques to minimize bias from residual confounding, reverse causation, and competing events.

We acknowledge several limitations. First, this was a retrospective study, and NAFLD was defined histologically; nevertheless, our case distribution, hazard estimates and absolute rate differences between histology categories accord with prior studies^8–12^ including a recent meta-analysis^5^, which argue against selection bias and underscore the generalizability of our results. Second, it is possible that the influence of NAFLD on cause-specific mortality may differ if NAFLD is diagnosed using non-invasive parameters; however, our findings are broadly consistent with prior population-based studies in which NAFLD was defined by ultrasound^41^ or administrative codes^24^. Third, pathology data may be subject to sampling error and inter-observer variability, and we lacked detailed data regarding the length and number of portal tracts in each biopsy; however, our validation study demonstrated the accuracy of our exposure definition, and we would emphasize that any nondifferential misclassification would most likely attenuate a true association. Fourth, despite careful matching and multivariable adjustment for clinical, demographic and medication confounders, residual confounding is possible, and we lacked detailed data regarding individual stages of non-cirrhotic fibrosis, smoking, alcohol consumption, body mass index [BMI], or laboratory values. However, our findings were robust in patients with and without clinical comorbidities, after re-matching NAFLD patients with full siblings, and after further accounting for incident alcohol abuse/misuse or a validated comorbidity index. Moreover, we demonstrated that an unmeasured confounder like BMI would need to be more strongly associated with mortality than previously described^42^ and also very highly imbalanced (i.e. both aHR≥4.5 and >50% difference between groups) to attenuate our results. Fifth, the Swedish population is primarily Caucasian, underscoring the need for research in diverse populations. Finally, although changing trends in NAFLD diagnostic strategies could have impacted our findings, all models accounted for calendar year, and our results were similar in recent time periods and in the NAFLD-only subgroup.

In conclusion, within a population-based cohort, all histological stages of NAFLD were associated with significantly increased risk of overall mortality, which increased in a dose-dependent manner with worsening NAFLD severity. Most of the excess mortality associated with NAFLD was from non-HCC cancer and cirrhosis, while in contrast, the contributions of cardiovascular disease and HCC were relatively modest. Our findings underscore the importance of reversing all stages of NAFLD, while also highlighting the need for effective public health strategies designed to prevent cancer and cirrhosis, in this high-risk and growing population.

## Supplementary Material

Supplementary Appendix

## Figures and Tables

**Table 1.** Characteristics of Adults with Histologically-Defined NAFLD and Matched Population Comparators, at the Index Date

Characteristic	Population Comparators *N=49,925*	All NAFLD *N=10,568*	Simple Steatosis *N=7,105*	NASH without Fibrosis *N=1,218*	Non-Cirrhotic Fibrosis *N=1,658*	Cirrhosis *N=587*
Female, %	45.4	44.8	43.9	48.6	46.0	44.5
Age at the index date, years (SD)	51.8 (14.5)	52.0 (14.5)	50.8 (14.6)	52.3 (14.9)	54.6 (13.9)	58.8 (11.6)
Years of follow-up, median (IQR)	16.8 (9.3–22.9)	14.2 (6.6–21.0)	16.4 (8.2–22.6)	12.8 (6.1–19.1)	9.3 (4.8–15.9)	7.8 (3.2–14.7)
Start of follow-up, %						
1966 – 1989	20.2	19.7	23.2	13.2	8.9	21.5
1990 – 2000	44.1	43.7	47.4	40.5	31.4	40.0
2001 – 2010	24.9	25.3	21.6	31.0	37.1	25.0
2011 – 2017	10.8	11.3	7.8	15.3	22.6	13.5
Nordic country of birth, %	91.5	89.7	90.4	88.0	87.7	89.8
Highest education level^1^, %	(among n=39,857)	(among n=8,482)	(among n=5,454)	(among n=1,057)	(among n=1,510)	(among n=461)
≤ 9 years	29.8	31.3	30.7	31.1	31.0	39.7
10–12 years	42.3	45.1	45.3	45.2	44.6	43.2
≥ 13 years	25.9	21..3	22.0	22.0	22.2	13.5
Unknown	2.1	2.3	2.1	2.1	2.2	3.7
Cardiovascular Disease, %	11.7	20.1	18.1	21.3	25.1	27.9
Dyslipidemia, %	3.8	7.1	4.7	9.4	13.8	11.6
Diabetes, %	2.8	11.2	8.2	12.6	18.2	25.9
Hypertension, %	4.8	9.8	6.3	12.2	19.5	19.1
Obesity, %	0.4	4.4	3.9	4.7	5.7	7.2
Metabolic syndrome^2^, %	0.7	2.8	1.5	4.4	5.4	8.5
Charlson Comorbidity Index^3^, mean (SD)	0.1 (0.6)	0.6 (1.4)	0.6 (1.4)	0.5 (1.4)	0.6 (1.4)	0.5 (1.4)

Abbreviations: SD, standard deviation; IQR, interquartile range; NAFLD, nonalcoholic fatty liver disease; NASH, nonalcoholic steatohepatitis

All variables reported as mean [SD] or %, unless described otherwise. For definitions of the NAFLD histological groups and all covariates, see the Appendix (eMethods).

1 Education categories based on compulsory school, high school, and college (see eMethods). Education level was recorded beginning in 1990, thus data presented are for persons with index dates on or after January 1, 1990. For all other analyses, persons with index dates prior to 1990 had education level recorded as missing.

2 Metabolic syndrome was defined as ≥3 metabolic risk factors (i.e. dyslipipidemia, diabetes, hypertension and/or obesity), as outlined in the Methods and in Table S2.

3 The calculation of the modified Charlson Comorbidity Index is outlined in the Supplementary Appendix (eMethods).

**Table 2.** All-Cause Mortality among Adults with Histologically-Confirmed NAFLD and Matched Population Comparators

		NAFLD*					
	Population Comparators N=49,925	All NAFLD N=10,568	Simple Steatosis N=7,105	NASH without Fibrosis N=1,218	Non-Cirrhotic Fibrosis N=1,658	Cirrhosis N=587	*P-trend*
							
Deaths, N.	13,911	4,338	2,823	478	637	400	--
Incidence Rate^1^ per 1000 PY [95% CI]	16.9 [16.6–17.2]	28.6 [27.8–29.5]	25.2 [24.3–26.2]	30.3 [27.7–33.1]	35.3 [32.6–38.1]	70.5 [63.9–77.5]	--
Absolute Rate Difference^1^, [95% CI]	0 [ref.]	11.7 [10.9–12.6]	8.3 [7.4–9.3]	13.4 [10.7–16.1]	18.4 [15.6–21.1]	53.6 [46.7–60.5]	--
20-Year Risk Difference^2^, % [95% CI]	0 [ref.]	15.3 [13.3–17.3]	10.7 [8.6–12.8]	18.5 [12.1–25.0]	25.6 [18.4–32.7]	49.4 [32.0–66.8]	--
Multivariable-adjusted HR^3^ [95% CI]	1 [ref.]	1.93 [1.86–2.00]	1.71 [1.64–1.79]	2.14 [1.93–2.38]	2.44 [2.22–2.69]	3.79 [3.34–4.30]	<0.01

Abbreviations: NAFLD, nonalcoholic fatty liver disease; NASH, nonalcoholic steatohepatitis; N., number; PY, person years; HR, hazard ratio; CI, confidence interval; ref., referent

* NAFLD was defined by liver histology. For definitions and algorithm, please see Methods and the Supplementary Appendix.

1 Confidence intervals for incidence rates and absolute rate differences were approximated by the normal distribution. Incidence rate difference is per 1000 person years.

2 20-year absolute risks and absolute risk differences [percentage points] were calculated based on Kaplan-Meier estimates.

3 The multivariable-adjusted model accounted for age at the index date, sex, county, calendar year, education level, cardiovascular disease, and the metabolic syndrome, defined as a composite categorical variable (ranging from 0 to 4) with 1 point given for each of the following conditions (i.e. diabetes, obesity, hypertension and/or dyslipidemia). For definitions, see Table S2.

**Table 3.** Cause-Specific Mortality among Adults with Histologically-Confirmed NAFLD* and Matched Population Comparators

		NAFLD*					
*Cause of Death, N.*	*Population Comparators*	*All NAFLD*	*Simple Steatosis*	*NASH without Fibrosis*	*Non-Cirrhotic Fibrosis*	*Cirrhosis*	*P for Trend*^4^
*Cancer*^1^	3,776	1,343	992	131	158	62	--
Incidence Rate^2^, per 1000 PY [95% CI]	4.8 [4.6–5.0]	9.3 [8.8–9.8]	9.2 [8.7–9.8]	8.7 [7.3–10.3]	9.3 [7.9–10.8]	11.3 [8.8–14.3]	--
Incidence Rate Difference^2^ [95% CI]	0 [ref.]	4.5 [3.9–5.0]	4.4 [3.8–5.0]	3.9 [2.4–5.4]	4.5 [3.0–5.9]	6.5 [3.7–9.3]	--
20-year Absolute Risk Difference^2^ [95% CI]	1 [ref.]	7.1 [6.0–8.2]	7.1 [5.9–8.4]	6.3 [3.0–9.6]	7.9 [4.3–11.5]	10.4 [3.5–17.2]	--
Multivariable-adjusted HR^3^ [95% CI]	1 [ref.]	2.16 [2.03–2.30]	2.17 [2.01–2.34]	2.08 [1.70–2.55]	2.26 [1.87–2.73]	2.12 [1.58–2.84]	0.09
*Cardiovascular Disease*	5,439	1,199	823	145	148	83	--
Incidence Rate^2^, per 1000 PY [95% CI]	6.9 [6.7–7.1]	8.3 [7.8–8.7]	7.6 [7.1–8.2]	9.6 [8.2–11.3]	8.7 [7.4–10.1]	15.1 [12.2–18.5]	--
Incidence Rate Difference^2^ [95% CI]	0 [ref.]	1.4 [0.9–1.9]	0.7 [0.2–1.3]	2.7 [1.2–4.3]	1.8 [0.4–3.2]	8.2 [5.0–11.5]	--
20-year Absolute Risk Difference^2^ [95% CI]	1 [ref.]	2.4 [1.2–3.5]	0.8 [−0.4–2.1]	5.2 [1.4–9.1]	5.7 [1.7–9.8]	16.9 [7.6–26.1]	--
Multivariable-adjusted HR^3^ [95% CI]	1 [ref.]	1.35 [1.26–1.44]	1.25 [1.16–1.35]	1.66 [1.38–2.01]	1.40 [1.17–1.69]	2.11 [1.63–2.73]	<0.01
*Cirrhosis*^1^	121	413	147	47	96	123	--
Incidence Rate^2^, per 1000 PY [95% CI]	0.2 [0.1–0.2]	2.8 [2.6–3.1]	1.4 [1.2–1.6]	3.1 [2.4–4.1]	5.6 [4.6–6.8]	22.4 [18.8–26.5]	--
Incidence Rate Difference^2^ [95% CI]	0 [ref.]	2.7 [2.4–2.8]	1.2 [1.0–1.4]	3.0 [2.1–3.9]	5.5 [4.4–6.6]	22.3 [18.3–26.2]	--
20-year Absolute Risk Difference^2^ [95% CI]	1 [ref.]	5.1 [4.5–5.7]	2.4 [1.9–2.9]	5.7 [3.7–7.7]	9.2 [6.9–11.5]	32.8 [24.6–41.1]	--
Multivariable-adjusted HR^3^ [95% CI]	1 [ref.]	18.15 [14.78–22.30]	9.29 [7.09–12.18]	28.29 [13.77–58.12]	26.03 [16.08–42.12]	166.25 [67.45–409.77]	<0.01
*Hepatocellular Carcinoma*^1^	96	186	88	22	45	31	--
Incidence Rate^2^, per 1000 PY [95% CI]	0.1 [0.1–0.2]	1.3 [1.1–1.5]	0.8 [0.7–1.0]	1.5 [1.0–2.1]	2.6 [2.0–3.5]	5.7 [4.0–7.8]	--
Incidence Rate Difference^2^ [95% CI]	0 [ref.]	1.2 [1.0–1.3]	0.7 [0.5–0.9]	1.3 [0.7–2.0]	2.5 [1.8–3.3]	5.5 [3.5–7.5]	--
20-year Absolute Risk Difference^2^ [95% CI]	1 [ref.]	2.2 [1.8–2.6]	1.2 [0.9–1.6]	3.0 [1.2–4.7]	5.3 [3.2–7.3]	12.3 [6.4–18.2]	--
Multivariable-adjusted HR^3^ [95% CI]	1 [ref.]	11.12 [8.65–14.30]	7.13 [5.18–9.83]	18.16 [7.9–41.6]	32.67 [15.15–70.45]	30.92 [14.30–66.87]	<0.01
*Other Causes*	3,685	1008	650	116	157	85	--
Incidence Rate^2^, per 1000 PY [95% CI]	4.7 [4.5–4.8]	6.9 [6.5–7.4]	6.0 [5.6–6.5]	7.7 [6.4–9.2]	9.2 [7.9–10.7]	15.5 [12.5–18.9]	--
Incidence Rate Difference^2^ [95% CI]	0 [ref.]	2.3 [1.8–2.7]	1.4 [0.9–1.9]	3.0 [1.6–4.4]	4.5 [3.1–6.0]	10.8 [7.5–14.1]	--
20-year Absolute Risk Difference^2^ [95% CI]	1 [ref.]	4.8 [3.8–5.9]	2.8 [1.7–4.0]	6.9 [3.3–10.5]	11.1 [6.9–15.2]	20.0 [11.1–29.0]	--
Multivariable-adjusted HR^3^ [95% CI]	1 [ref.]	1.75 [1.63–1.87]	1.55 [1.42–1.69]	2.06 [1.67–2.55]	2.28 [1.89–2.76]	2.91 [2.25–3.78]	<0.01

Abbreviations: NAFLD, nonalcoholic fatty liver disease; NASH, nonalcoholic steatohepatitis; HCC, hepatocellular carcinoma; N., number; PY, person years; HR, hazard ratio; CI, confidence interval

* NAFLD was defined from liver histology, as outlined in the Methods and Supplementary Methods. The analyses of cause-specific mortality contain fewer subjects than the analyses of all-cause mortality, because the end of follow-up for the Cause of Death Register was December 31, 2016.

1 Because HCC-specific mortality was assessed separately, cancer-specific mortality included deaths from all cancers except HCC; similarly, cirrhosis-specific mortality encompassed deaths from all non-HCC related complications of chronic liver disease (for details, see eMethods).

2 Incidence rate differences per 1000 person-years. Confidence intervals for incidence rates and absolute rate differences were approximated by the normal distribution.

20-year absolute risks and risk differences [percentage points] were calculated based on Kaplan-Meier estimates.

3 The multivariable model accounted for the covariates outlined in the footnotes to Table 2.

4 P for linear trend was estimated across NAFLD histology categories (modeled continuously), compared to population comparators; for details, see Methods.

**Table 4.** Risk of All-Cause Mortality in the NAFLD-only Subgroup*

	Simple Steatosis (ref.)* N=7,105	NASH without Fibrosis* N=1,218	Non-Cirrhotic Fibrosis* N=1,658	Cirrhosis* N=587	P for trend^4^
Deaths, N.	2,823	478	637	400	--
Incidence Rate^1^, per 1000 PY [95% CI]	25.2 [24.3–26.2]	30.3 [27.7–33.1]	35.3 [32.6–38.1]	70.5 [63.9–77.5]	--
Incidence Rate Difference^1^ [95% CI]	0 [ref.]	5.1 [2.2–7.9]	10.0 [7.2–12.9]	45.3 [38.3–52.2]	--
20-Year Absolute Risk Difference^2^, % [95% CI]	0 [ref.]	7.9 [1.1–14.6]	14.9 [7.5–22.3]	38.7 [21.2–56.2]	--
Multivariable-adjusted HR^3^ [95% CI]	1 [ref.]	1.14 [1.03–1.26]	1.26 [1.15–1.38]	1.95 [1.75–2.18]	< 0.01

Abbreviations: NAFLD, nonalcoholic fatty liver disease; NASH, nonalcoholic steatohepatitis; N., number; PY, person years; HR, hazard ratio; CI, confidence interval; ref., referent

* NAFLD was defined by liver histology as outlined in the Supplementary eMethods.

1 Confidence intervals for incidence rates and absolute rate differences were approximated by the normal distribution.

2 20-year absolute risks and risk differences (percentage points) were calculated based on Kaplan-Meier estimates.

3 The multivariable model accounted for the covariates outlined in the footnotes to Table 2.

4 P for linear trend was estimated across NAFLD histology categories (modeled continuously), compared to simple steatosis; for details, see Methods.

## Summary Box

### What is already known about this subject?

- Nonalcoholic fatty liver disease (NAFLD) with advanced fibrosis is associated with an increased risk of overall and liver-specific mortality.
- However, the risk of overall and cause-specific mortality across the full histologic spectrum of NAFLD has yet to be established.

### What are the new findings?

- Among adults in Sweden with biopsy-confirmed NAFLD, the overall mortality rate was significantly elevated, compared to age, sex, county and calendar year matched participants without NAFLD.
- Significant excess mortality risk was found across all stages of NAFLD, and it increased progressively with worsening NAFLD severity.
- This increased risk was primarily due to deaths from extra-hepatic cancer and cirrhosis, while in contrast, the contributions of cardiovascular disease and hepatocellular carcinoma were relatively modest.

### How might it impact on clinical practice in the foreseeable future?

- These findings underscore the importance of reversing all stages of NAFLD.
- Public health efforts focused on the prevention of cancer and cirrhosis in patients with NAFLD should be prioritized.

## List of Abbreviations:

aHR: Adjusted hazard ratio
ARD: Absolute rate difference
CI: Confidence interval
ESPRESSO: Epidemiology Strengthened by histoPathology Reports in Sweden
HCC: Hepatocellular carcinoma
ICD: International classification of diseases
LISA: Longitudinal integrated database for health insurance and labour market studies
Mg: Milligrams
NAFLD: Nonalcoholic fatty liver disease
NASH: Nonalcoholic steatohepatitis
PIN: Personal identity number
PY: Person-Years
PPV: Positive predictive value
SNOMED: Systematized Nomenclature of Medicine
