# Supplementary material for: Mortality in Biopsy-Confirmed Nonalcoholic Fatty Liver Disease: Results From A Nationwide Cohort
Source: Gut. Author manuscript; Available in PMC 2021 Jul 1. (PMC8185553; doi:10.1136/gutjnl-2020-322786)
Supplement: Supplementary Appendix [file NIHMS1709634-supplement-Supplementary_Appendix.pdf]

Supplementary Appendix

eMethods

- Definition of NAFLD and NAFLD Histological Categories
- Definitions of Secondary Outcomes
- Definitions of Covariates and Construction of Multivariable Models
- Missing Data
- Construction of the Modified Charlson Comorbidity Index

eTables

- **Table S1.** Study Exclusion Criteria
- **Table S2.** Definitions of Covariates and Prescription Medications
- **Table S3.** Cause-Specific Mortality among NAFLD Patients and Matched Population Comparators, after Accounting for Competing Risks
- **Table S4.** Cause-Specific Mortality in the NAFLD-only Subgroup
- **Table S5.** All-Cause Mortality among Adults with NAFLD and Matched Unaffected Siblings
- **Table S6.** All-Cause Mortality Among Adults with NAFLD and Population Comparators, 2006 - 2017
- **Table S7A.** All-cause Mortality among Adults with NAFLD and Population Comparators, after Further Adjustment for the Modified Charlson Comorbidity Index
- **Table S7B.** All-cause Mortality in the NAFLD-only Sub-group, after Further Adjustment for the Modified Charlson Comorbidity Index
- **Table S8**. Array-Approach Sensitivity Analysis for an Unmeasured Confounder

Supplementary Figures

- **Figure S1.** Construction of the Nationwide Matched Cohort
- **Figure S2.** NAFLD and Risk of All-Cause Mortality According to Selected Strata

Supplementary References

eMethods:

Definition of NAFLD and NAFLD Histological Categories

Consistent with nationwide liver histopathology reporting recommendations provided to all pathologists in Sweden[1], we defined NAFLD from histopathology reports using an established algorithm of SNOMED topography and morphology codes, after excluding other etiologies of liver disease. Specifically, patients were identified by a liver biopsy histopathology report that included a topography code for liver (T56), and at least one morphology code for steatosis (M5008x or M5520x), consistent with our validation study in ESPRESSO (see below). In Sweden, clinically-indicated liver biopsies are conducted with a single pass of the liver, unless a satisfactory specimen is not obtained with the initial pass. Furthermore, consistent with Swedish liver histopathology reporting recommendations, it is documented if any biopsy is too short (i.e. <15mm in length), contains fewer than 5 portal tracts, or is fragmented[1].

Among individuals with multiple liver biopsies that demonstrated steatosis, we included the first such biopsy. We excluded anyone with at least one primary diagnosis or the second of two secondary diagnoses of another etiology of liver disease (all outlined in **Table S1**; i.e. alcohol-related liver disease, drug-induced liver disease, viral hepatitis B or C infection, Budd-Chiari, liver abscess, HIV/AIDS, autoimmune hepatitis, primary biliary cholangitis, other cholangitis, alpha-1 antitrypsin deficiency, glycogen storage disease), or a primary diagnosis or the second or two secondary diagnoses of alcohol abuse/misuse or drug abuse. Additionally, we further excluded anyone with use of a steatogenic medication or a medication specifically used to treat an alternative etiology of liver disease (as per **Table S1**). Medication use was defined by the Prescribed Drug Register as a filled prescription for at least 30 cumulative defined daily doses (cDDD) of a given medication, at any time prior to the index date (or corresponding matching date), with the exception of systemic steroids (for which a person was excluded if they used systemic steroids within 0 to 3 months prior to the index date).

All patients who met our criteria for NAFLD were further classified at the time of their index biopsy into one of 4 mutually exclusive categories of histological severity (i.e. simple steatosis, NASH

without fibrosis, non-cirrhotic fibrosis and cirrhosis), using validated definitions (see Methods for our validation study). Specifically, simple steatosis was defined by at least 1 code for steatosis and no additional codes for inflammation (i.e. M5400x or M4-) or fibrosis (i.e. M4900x) or cirrhosis (i.e. M4950x). NASH without fibrosis was defined broadly by the presence of at least 1 code for steatosis plus at least 1 code for inflammation (i.e. M5400x or M4-), without any codes for fibrosis or cirrhosis. Non-cirrhotic fibrosis (i.e. F1-F3 fibrosis, with or without NASH) was defined by the presence of at least 1 code for steatosis plus at least 1 code for fibrosis (i.e. M4900x), but no codes for cirrhosis. Cirrhosis was defined by at least 1 code for cirrhosis (i.e. M4950x).

Definitions of Secondary Outcomes

The primary outcome was all-cause mortality (see Methods for details). Secondary outcomes included specific causes of mortality, ascertained from the Cause of Death Register. We categorized cause-specific mortality in 5 groups. Cardiovascular-specific mortality was defined from the Cause of Death Register by a primary ICD code for cardiovascular disease (ICD-8 / 9: 390-459; ICD-10: I01-I99). Hepatocellular carcinoma (HCC)-specific mortality was defined from the Cause of Death Register by a primary ICD code for HCC (ICD-7: 155.0, 155,0, 1550; ICD9: 155A, 155.0; ICD-10: C22.0, C22.9, C22.99). Cirrhosis-specific mortality was defined as death from any non-HCC cause of liver disease or liver failure, defined by a primary ICD code for cirrhosis, liver failure, decompensated liver disease (i.e. variceal bleeding, spontaneous bacterial peritonitis, encephalopathy or hepatorenal syndrome), or death from liver transplantation, as outlined in **Table S2**. Cancer-specific mortality was defined as death from any non-HCC cancers, defined from the Cause of Death Register by a primary ICD code for any cancer (ICD7 / 8: 140-239; ICD10: C00-C99; with the exclusion of any HCC-specific ICD code). All other deaths were classified as, “other causes of mortality”.

Definitions of Covariates and Construction of Multivariable Models

Definitions of clinical, demographic and prescription medication covariates are outlined in the **Methods** and in **Table S2**. Since 1990, the longitudinal integrated database for health insurance and labour market studies (LISA) database[2] has prospectively recorded and annually-updated

detailed data from the Swedish labor market, and also from the educational and social sectors, for all Swedish residents aged 16 years and older. Education level, a proxy for socioeconomic status, was obtained from the validated LISA database[2].

For the primary analysis, our main multivariable model adjusted for matching factors (i.e. age at the index date/corresponding matching date, sex, calendar year and county in Sweden) as well as the following *a priori* defined prognostic covariates: education level, cardiovascular disease, and the components of the metabolic syndrome (i.e. diabetes, obesity, hypertension and dyslipidemia). We also conducted a series of sensitivity analyses, to further address potential residual confounding. In one analysis, we repeated the primary analysis after restricting the cohort to persons with an index date on or after January 1, 2006, and then constructed an additional multivariable-adjusted model that further accounted for the following *a priori* time-varying medication covariates: low-dose aspirin (<163mg dosage), statin medications, metformin, other antidiabetic medications and anti-hypertensive medications (as outlined in **Table S2**). For each medication, use was defined from the Prescription Drug Register, as at least 30 cumulative defined daily doses (cDDD) of filled prescriptions for that medication from a Swedish pharmacy, and this information was updated over each monthly interval of study follow-up.

In an additional sensitivity analysis, we constructed a separate multivariable model that further adjusted for a modified Charlson Comorbidity Index (CCI) score. This modified CCI score was constructed specifically to incorporate known or putative confounders not previously included in our main multivariable model, and thereby further address potential residual confounding. This modified CCI score included: Chronic Obstructive Pulmonary Disease (COPD), rheumatic or connective tissue disease (i.e. lupus, polymyalgia rheumatica, polymyositis, mixed connective tissue disease, and/or moderate-to-severe rheumatoid arthritis), Peptic ulcer disease, Hemiplegia, moderate-to-severe chronic kidney disease (CKD), solid tumor, Leukemia and Lymphoma. We excluded conditions that already were accounted for in the main multivariable model (i.e. age, myocardial infarction, congestive heart failure, peripheral vascular disease, stroke or transient ischemic attack). We also did not include acquired immune deficiency syndrome (AIDS), because

we previously had excluded anyone with HIV/AIDS from this analysis. Finally, because liver disease severity could represent intermediates that lie on the causal pathway between NAFLD and the study outcome (mortality), and because those factors were already accounted for by our assessments of NAFLD histological severity, we did not include them in the modified CCI Score.

Missing Data

Data were missing for education level in <2.5% of adults with an index date on or after January 1, 1990, which was the first year in which the LISA database was available[2]. This included 2.1% of population comparators and 2.3% of NAFLD patients, as outlined in Table 1. For our multivariable models, we included a separate missing category for missingness on education level.

The nationwide Swedish registers provide complete information on liver biopsies and deaths including cause of death, as mandated by the government, and the validity and completeness of clinical comorbidities are well-established (see Methods). Despite this, missing data do arise, primarily from under-reporting. For example, if an individual does not have a coded diagnosis for obesity, this would be classified as “no obesity”, however the absence of a diagnostic code for obesity could be due to either (1) a true negative (i.e. the individual does not have obesity), or (2) obesity was not recorded. Thus, consistent with prior work[3], no other data were considered missing, with the sole exception of education level.

Table S1. Study Exclusion Criteria*

Excluded Conditions[1]:	ICD-7 / 8	ICD-9	ICD-10
Alcohol abuse / misuse, or Alcohol-related liver disease	280,00; 281,00; 307,00; 307,10; 307,99; 322; 581,10; 583,10; 261,00; 262,00; 291; 291,1; 303; 571,00; 571,01; 979; 980,00; 980,01; 980,98; 980,99	291; 294A; 303; 305A; 357F; 425F; 535D; 571A-D; 760W; 790D; 977D; 980A; 980X; V97B	E24.4; F10; G31.2; G62.1; G72.1; I42.6; K29.2; K70; K86.0; Q35.4; R78.0; T51.0; T51.8; T51.9; X65; Y15; Y57.3; Y90; Y91; Z50.2; Z71.4; Z71.2
Other drug abuse	5710, E860, N980	571A-D	F11-F19
Drug-induced liver disease		573D	K71
Viral hepatitis B or C infection		70; 070; ICD-8: 070; 999,20	B15-19, B16.0, B16.1, B16.2, B16.9, B17.0-B17.9, B18.0-B18.9, B19.0-B19.9; B00.8; B25.1
Budd-Chiari		453A	I82
Liver abscess	572; 572,00	5720	K75.0, A06.4
HIV		279K	B20-B24
Hemochromatosis	273,2	275A	E83.1
Wilson's disease	273,3	275B	E83.0
Autoimmune hepatitis		571.42	K75.4
Primary biliary cholangitis		571G	K74.3, K74.4
Other cholangitis	574,06	576B	K83; K83.0A
Alpha-1 antitrypsin deficiency		273.4; 277G; 274E; 573W	E88.01
Glycogen storage disease		271.8	E74.09, E74.00

**Excluded Medications[2]:**	**Prescribed Drug Register ATC Code:**
Systemic steroids	01AC02, C05AA09, D07AB19, D10AA03, H02AB02, R01AD03, S01BA01, S01CB01, S02BA06, S03BA01, H02AB15, A01AC03, A07EA02, C05AA01, D07AA02, D07XA01, H02AB09, S01BA02, S01CB03, S02BA01, D07AC16, D07AB11, D07AB02, D07AC16, A07AE03, H02AB07
Tamoxifen	L02BA01
Methotrexate	L01BA01, L04AX03
Interferon	L03AB
Direct-acting antiviral therapy	JO5, JO5A, JO5AA-AH, JO5AP, JO5AR
Nucleos(t)ide reverse transcriptase inhibitors / NNRTIs	J05AF, J05AG
Valproic acid	N03AG01
Amiodarone	C01BD01

Abbreviations: ICD, International Classification of Disease; ATC, anatomic therapeutic chemical classification
*For study flowchart outlining exclusion criteria, please see Figure S1
[1] We excluded any person with a first recorded primary diagnosis or the second of two secondary diagnoses for another etiology of liver disease, or alcohol abuse/misuse or alcohol-related liver disease, defined on or prior to the index date.
[2]We excluded any person with use of a steatogenic medication or a medication used to treat an alternative etiology of liver disease. Medication use was defined by a filled prescription for at least 30 cumulative defined daily doses (cDDD), in the Prescribed Drug Register, at any time prior to the index date (or corresponding matching date), with the exception of systemic steroids (for which a person was excluded if they used systemic steroids within 0 to 3 months prior to the index date). As the Prescribed Drug Register began on July 1, 2005, these exclusion criteria were only applied to the subgroup analysis in which the cohort was restricted to persons with an index biopsy (or corresponding matching date) on or after January 1, 2006.

Table S2. Definitions of Covariates and Prescription Medications

Entity	Source	Definition[a]
Low-dose aspirin[c]	Prescribed Drug Register[b]	Medication ATC code B01AC06
Statins	Prescribed Drug Register	Medication ATC codes: C10
Metformin	Prescribed Drug Register	Medication ATC code: A10BA02
Insulin	Prescribed Drug Register	Medication ATC codes: A10A
Other glucose-lowering agents (not Metformin or Insulin)	Prescribed Drug Register	Medication ATC codes: A10B (excluding A10BA02)
Antihypertensive medications	Prescribed Drug Register	Medication ATC codes: C02A-N, C03AA, C03AB, C03BA, C03CA, C03DA, C03EA, C08DA51, C08DA, C08CA, C08DA, C08DB, C09AB, C09BA, C09BB, C09CA, C093CB, C09DA, C09DB01
Oral anticoagulants	Prescribed Drug Register	Medication ATC codes: B01AA
Coronary vasodilators	Prescribed Drug Register	Medication ATC codes: C07AA, C07AB, C07AB02, C07BB, C07CB, C07AG01
Liver transplantation	Hospital discharge letters, using the Swedish Classification of Operation and Major Procedures codes	Codes: 5200, 5202, JJC00, JJC10, JJC20, JJC30, JJC40
Cardiovascular disease	Hospital discharge letters or outpatient specialty care letters (Patient Register) for coronary heart disease, arrhythmia, vascular disease, congestive heart failure, and/or receipt of at least one 30-day filled prescription for a cardiovascular-specific oral anticoagulant or coronary vasodilator medication (Prescribed Drug Register)	(See individual definitions of each component, below)

Entity	Source	Definition[a]
Coronary heart disease	Hospital discharge letters or outpatient specialty care letters	ICD-8: 410-411<br>ICD-9: 413<br>ICD-10: I11, I20-25, I65-66, I70-74
Arrhythmia	Hospital discharge letters or outpatient specialty care letters	ICD-8: 427.20-427.97; ICD-9 427D;<br>ICD-10: I44-49
Vascular disease	Hospital discharge letters or outpatient specialty care letters	Stroke or TIA (ICD-8/9 430-438; ICD-10 G45-46, I60-69); peripheral vascular disease (ICD-8 432, 440-442, 444-445, 447; ICD-9 443.9; ICD-10: I65, I70-77, K55.0-55.1)
Congestive heart failure	Hospital discharge letters or outpatient specialty care letters	ICD-8: 402, 404, 425, 42709-42719, 428; ICD-9: 425, 428<br>ICD-10: I1110, I130, I42, I43, I50, J81
Metabolic syndrome	At least 3 or more of the following criteria: Hypertension, Diabetes, Obesity and/or Dyslipidemia (defined below)	(See individual definitions of each component, below)
Hypertension	Hospital discharge letters or outpatient specialty care letters (Patient Register) and/or receipt of at least one 30-day filled prescription for an anti-hypertensive drug (Prescribed Drug Register)	ICD-9: 400-404<br>ICD-9: 401-405<br>ICD-10: I10-I16<br>Medication ATC codes: (see antihypertensives, above)
Diabetes	Hospital discharge letters containing either a primary or a secondary diagnosis for diabetes or outpatient specialty care diagnosis (Patient Register), and/or at least one 30-day filled prescription for an antidiabetic agent (Prescribed Drug Register)	ICD-8 / ICD-9: 250<br>ICD-10: E10.0-E14.9<br>Medication ATC Codes: A10A (insulin), A10BA (biguanides), A10BB-BX (other glucose-lowering drugs)
Obesity	Hospital discharge letters or outpatient specialty care letters	ICD-8: 278, 649,1<br>ICD-9: 278, 649B<br>ICD-10: E65-66

Entity	Source	Definition[a]
Dyslipidemia	Hospital discharge letters or outpatient specialty care letters (Patient Register) and/or receipt of at least one 30-day filled prescription for a statin medication (Prescribed Drug Register)	ICD-8: 272<br>ICD-9: 272<br>ICD-10: E78<br>Medication ATC codes: C10

Abbreviations: ICD, International Classification of Diseases; ATC, Anatomical Therapeutic Chemical classification system; TIA, transient ischemic attack

[a]Covariates were defined using ICD classifications. Note that ICD 4th letter codes were used in the Swedish versions of ICD, and they have the same meaning as the 4th digit number used in the US version of ICD

[b]The Prescribed Drug Register includes dates of prescriptions and dispensations, defined daily doses (DDD) of all drug prescriptions and dispensations, according to the Anatomical Therapeutic Chemical (ATC) Classification System. For covariates that included medications, relevant medication use was defined as the first filled prescription for >30 cDDD.

[c]Low-dose aspirin prescriptions in Sweden include strengths of <163mg (typically either 75mg or 160mg)

Table S3. Cause-Specific Mortality among NAFLD Patients and Matched Population Comparators, after Accounting for Competing Risks*

**Cause of Death***	*Population Comparators*	**NAFLD**					
		All NAFLD	*Simple Steatosis*	*NASH without Fibrosis*	*Non-Cirrhotic Fibrosis*	*Cirrhosis*	*P for trend*[2]
Cancer*, aHR[1] [95% CI]	1 [ref.]	1.84 [1.72-1.96]	1.94 [1.81-2.09]	1.70 [1.39-2.08]	1.80 [1.50-2.17]	1.20 [0.90-1.60]	<0.01
Cardiovascular Disease, aHR[1] [95% CI]	1 [ref.]	0.98 [0.92-1.04]	0.93 [0.87-1.01]	1.26 [1.04-1.51]	1.00 [0.83-1.20]	1.02 [0.79-1.31]	0.75
Liver Disease*, aHR[1] [95% CI]	1 [ref.]	1.74 [1.68-1.80]	1.52 [1.46-1.59]	1.94 [1.74-2.17]	2.27 [2.06-2.50]	3.60 [3.17-4.09]	<0.01
HCC*, aHR[1] [95% CI]	1 [ref.]	8.67 [6.75-11.14]	5.83 [4.24-8.03]	12.49 [5.51-28.31]	23.81 [11.11-51.00]	15.26 [7.27-32.06]	<0.01

Abbreviations: HCC, hepatocellular carcinoma; NAFLD, nonalcoholic fatty liver disease; NASH, nonalcoholic steatohepatitis; aHR, adjusted hazard ratio; CI, confidence interval; ref., referent
*For each specific cause of death, competing events included death from any other cause, as outlined in the Methods. Because HCC-specific mortality was assessed separately, cancer mortality did not include deaths from HCC; similarly, cirrhosis-related mortality also did not include deaths from HCC (for details, see eMethods).
[1]For each analysis, the multivariable-adjusted subdistribution hazards regression model accounted for competing risks of other causes of death, and adjusted for the covariates outlined in the footnotes to Table 2.
[2]P for linear trend was estimated across NAFLD histology categories (modeled continuously), compared to population comparators; for details, see Methods.

Table S4. Cause-Specific Mortality in the NAFLD-only Subgroup*

Cause of Death	*Simple Steatosis** *N=7,105*	*NASH without Fibrosis** *N=1,218*	*Non-Cirrhotic Fibrosis** *N=1,658*	*Cirrhosis** *N=587*	*P for trend*[4]
Cancer[1], *N.*	992	131	158	62	--
Incidence Rate[1], per 1000 PY [95% CI]	9.2 [8.7-9.8]	8.7 [7.3-10.3]	9.3 [7.9-10.8]	11.3 [8.8-14.3]	--
20-year Absolute Risk[2], % [95% CI]	15.9 [15.1-16.8]	15.1 [12.8-17.5]	16.7 [14.2-19.3]	19.2 [14.3-24.1]	--
20-Year Risk Difference[2], % [95% CI]	0 [ref.]	-0.8 [-4.3-2.7]	0.8 [-3.0-4.5]	3.3 [-3.6-10.2]	--
Multivariable-adjusted HR[3] [95% CI]	1 [ref.]	0.79 [0.65-0.95]	0.75 [0.63-0.89]	0.79 [0.61-1.03]	0.87
Cardiovascular Disease, N.	823	145	148	83	--
Incidence Rate[1], per 1000 PY [95% CI]	7.6 [7.1-8.2]	9.6 [8.2-11.3]	8.7 [7.4-10.1]	15.1 [12.2-18.5]	--
20-year Absolute Risk[2], % [95% CI]	13.1 [12.2-13.9]	17.4 [14.7-20.2]	18.0 [15.1-20.7=8]	29.1 [22.5-35.7]	--
20-Year Risk Difference[2], % [95% CI]	0 [ref.]	4.4 [0.4-8.4]	4.9 [0.8-9.0]	16.0 [6.7-25.3]	--
Multivariable-adjusted HR[3] [95% CI]	1 [ref.]	1.28 [1.07-1.53]	1.18 [0.98-1.43]	1.46 [1.16-1.84]	0.50
Liver Disease[1], *N.*	147	47	96	123	--
Incidence Rate[1], per 1000 PY [95% CI]	1.4 [1.2-1.6]	3.1 [2.4-4.1]	5.6 [4.6-6.8]	22.4 [18.8-26.5]	--
20-year Absolute Risk[2], % [95% CI]	2.7 [2.3-3.1]	6.0 [4.6-7.4]	9.5 [7.8-11.2]	33.1 [27.3-39.0]	--
20-Year Risk Difference[2], % [95% CI]	0 [ref.]	3.3 [1.3-5.3]	6.8 [4.4-9.2]	30.4 [22.2-38.7]	--
Multivariable-adjusted HR[3] [95% CI]	1 [ref.]	2.19 [1.55-3.08]	3.76 [2.83-4.99]	13.21 [10.17-17.16]	<0.01
Hepatocellular Carcinoma[1], *N.*	88	22	45	31	--
Incidence Rate[1], per 1000 PY [95% CI]	0.8 [0.7-1.0]	1.5 [1.0-2.1]	2.6 [2.0-3.5]	5.7 [4.0-7.8]	--
20-year Absolute Risk[2], % [95% CI]	1.4 [1.2-1.7]	3.2 [1.9-4.4]	5.5 [4.0-6.9]	12.5 [8.3-16.7]	--
20-Year Risk Difference[2], % [95% CI]	0 [ref.]	1.7 [0.0-3.5]	4.0 [2.0-6.1]	11.1 [5.2-17.0]	--
Multivariable-adjusted HR[3] [95% CI]	1 [ref.]	1.76 [1.08-2.88]	2.67 [1.79-3.97]	4.51 [2.90-7.03]	<0.01
Other Causes, N.	650	116	157	85	--
Incidence Rate[1], per 1000 PY [95% CI]	6.0 [5.6-6.5]	7.7 [6.4-9.2]	9.2 [7.9-10.7]	15.5 [12.5-18.9]	--
20-year Absolute Risk[2], % [95% CI]	11.1 [10.3-11.9]	15.2 [12.6-17.7]	19.3 [16.4-22.3]	28.3 [21.9-34.7]	--
20-Year Risk Difference[2], % [95% CI]	0 [ref.]	4.1 [0.4-7.8]	8.2 [3.9-12.4]	17.2 [8.2-26.2]	--
Multivariable-adjusted HR[3] [95% CI]	1 [ref.]	1.28 [1.04-1.57]	1.53 [1.27-1.84]	1.91 [1.51-2.42]	<0.01

Abbreviations: NAFLD, nonalcoholic fatty liver disease; NASH, nonalcoholic steatohepatitis; HCC, hepatocellular carcinoma; N., number; PY, person years; HR, hazard ratio; CI, confidence interval; ref., referent

*NAFLD was defined from liver histology, as outlined in the Methods and Supplementary Methods. The analyses of cause-specific mortality contain fewer subjects than the analyses of all-cause mortality, because the end of follow-up for the Cause of Death Register was December 31, 2016.

[1]Because HCC-specific mortality was assessed separately, cancer-specific mortality included deaths from all cancers except HCC; similarly, liver-specific mortality encompassed deaths from all non-HCC causes of liver disease (for details, see eMethods).

[2]Confidence intervals for incidence rates and absolute rate differences were approximated by the normal distribution. 20-year absolute risks and risk differences [percentage points] were calculated based on Kaplan-Meier estimates.

[3] The multivariable-adjusted model accounted for the covariates outlined in the footnotes to Table 2.

[4]P for linear trend was estimated across NAFLD histology categories (modeled continuously), compared to simple steatosis; for details, see Methods.

Table S5. All-Cause Mortality Among Adults with NAFLD* and Matched Unaffected Siblings

		NAFLD*					
	Unaffected Siblings N=9,554	*All NAFLD N=4,850*	*Simple Steatosis N=3,282*	*NASH without Fibrosis N=557*	*Non-Cirrhotic Fibrosis N=788*	*Cirrhosis N=223*	*P for trend[4]*
Deaths, N.	1118	1164	713	124	207	120	--
Incidence Rate[1], per 1000 PY [95% CI]	6.9 [6.5-7.3]	15.6 [14.7-16.6]	12.8 [11.9-13.8]	16.7 [13.8-19.9]	23.0 [20.0-26.3]	54.5 [45.2-65.2]	--
Incidence Rate Difference[1], [95% CI]	0 [ref.]	8.7 [7.8-9.7]	5.9 [4.9-6.9]	9.75 [6.8-12.7]	16.1 [12.9-19.3]	47.7 [37.9-57.4]	--
20-Year Risk Difference[2], % [95% CI]	0 [ref.]	12.8 [10.7-15.0]	8.1 [6.0-10.3]	13.8 [7.4-20.2]	25.6 [17.6-33.7]	54.8 [30.6-79.0]	--
Multivariable-adjusted HR[3] [95% CI]	1 [ref.]	2.47 [2.23-2.73]	2.01 [1.78-2.27]	3.49 [2.51-4.86]	3.48 [2.67-4.53]	6.32 [3.97-10.07]	<0.01

Abbreviations: NAFLD, nonalcoholic fatty liver disease; NASH, nonalcoholic steatohepatitis; N., number; PY, person years; HR, hazard ratio; CI, confidence interval; ref., referent
*NAFLD was defined by liver histology. For definitions and algorithm, see Methods and the Appendix. Incidence rate differences are expressed per 1000 person-years.
[1]Confidence intervals for incidence rates and absolute rate differences were approximated by the normal distribution.
[2]20-year absolute risks and risk differences [percentage points] were calculated based on Kaplan-Meier estimates.
[3] The multivariable-adjusted model was stratified by family [i.e. 1 stratum per family] and accounted for age at the index date, sex, county, calendar year, education level, cardiovascular disease, and the components of the metabolic syndrome [i.e. diabetes, obesity, hypertension, dyslipidemia] at the index date.
[4]P for linear trend was estimated across NAFLD histology categories (modeled continuously), compared to matched unaffected full siblings; for details, see Methods.

Table S6. All-Cause Mortality Among Adults with NAFLD and Population Comparators, 2006 - 2017

		NAFLD*					
	Population Comparators N=10,856	*All NAFLD N=2,375*	*Simple Steatosis N=1,163*	*NASH without Fibrosis N=347*	*Non-Cirrhotic Fibrosis N=702*	*Cirrhosis N=163*	*P for trend[4]*
Deaths, N.	621	420	182	50	123	65	--
Incidence Rate[1], per 1000 PY [95% CI]	8.8 [8.1-9.5]	29.9 [27.2-32.8]	25.2 [21.8-29.0]	24.3 [18.5-31.5]	30.8 [25.8-36.4]	82.6 [64.9-103.8]	--
Incidence Rate Difference[1], [95% CI]	0 [ref.]	21.1 [18.2-24.0]	16.4 [12.7-20.2]	15.6 [8.8-22.3]	22.0 [16.5-27.5]	73.8 [53.7-93.9]	--
10-Year Risk Difference[2], % [95% CI]	0 [ref.]	15.7 [12.5-19.0]	11.0 [7.3-14.8]	12.9 [5.1-20.7]	19.0 [12.1-25.8]	45.5 [19.0-71.9]	--
Multivariable-adjusted HR[3] [95% CI]	1 [ref.]	3.52 [3.09-4.00]	3.25 [2.68-3.93]	2.94 [2.04-4.23]	3.47 [2.73-4.41]	7.74 [5.04-11.89]	<0.01

Abbreviations: NAFLD, nonalcoholic fatty liver disease; NASH, nonalcoholic steatohepatitis; N., number; PY, person years; HR, hazard ratio; CI, confidence interval; ref., referent

*NAFLD was defined by liver histology. For definitions and algorithm, please see Methods and the Supplementary Appendix. Incidence rates and incidence rate differences are reported per 1000 person-years.

[11]Confidence intervals for incidence rates and absolute rate differences were approximated by the normal distribution.

[2]10-year absolute risks and risk differences [percentage points] were calculated based on Kaplan-Meier estimates.

[3] The multivariable-adjusted model accounted for age at the index date, sex, county, calendar year, education level, cardiovascular disease, and the components of the metabolic syndrome [i.e. diabetes, obesity, hypertension, dyslipidemia] at the index date, and further adjusted for time-varying medication covariates, updated over each month of follow-up, as outlined in the Supplementary Methods.

[4]P for linear trend was estimated across NAFLD histology categories (modeled continuously), compared to population comparators; for details, see Methods.

Table S7A. All-cause Mortality Among Adults with NAFLD and Matched Population Comparators, after Further Adjustment for the Modified Charlson Comorbidity Index (CCI)*

		NAFLD					
	Population Comparators N=49,925	*All NAFLD N=10,568*	*Simple Steatosis N=7,105*	*NASH without Fibrosis N=1,218*	*Non-Cirrhotic Fibrosis N=1,658*	*Cirrhosis N=587*	*P-trend*[2]
Multivariable aHR[1] [95% CI] – *from Table 2*	1 [ref.]	1.93 [1.86-2.00]	1.71 [1.64-1.79]	2.14 [1.93-2.38]	2.44 [2.22-2.69]	3.79 [3.34-4.30]	<0.01
Multivariable aHR[1] + Modified CCI [95% CI]	1 [ref.]	1.74 [1.68-1.80]	1.52 [1.46-1.59]	1.94 [1.74-2.17]	2.27 [2.06-2.50]	3.60 [3.17-4.09]	<0.01

Abbreviations: NAFLD, nonalcoholic fatty liver disease; NASH, nonalcoholic steatohepatitis; aHR, adjusted hazard ratio; CI, confidence interval; ref., referent
*The construction of the modified Charlson Comorbidity Index [CCI] is outlined in the Supplementary Methods.
[1]The multivariable-adjusted model accounted for the covariates outlined in the footnotes to Table 2, plus the modified Charlson Comorbidity Index [CCI].
[2]P for linear trend was estimated across NAFLD histology categories (modeled continuously), compared to population comparators; for details, see Methods

Table S7B. All-cause Mortality in the NAFLD-Only Subgroup, after Further Adjustment for the Modified Charlson Comorbidity Index* (CCI)

	Simple Steatosis [ref.] *N=7,105*	*NASH without Fibrosis* *N=1,218*	*Non-Cirrhotic Fibrosis* *N=1,658*	*Cirrhosis* *N=587*	*P-trend*[2]
Multivariable aHR[1] [95% CI] – *from Table 4*	1 [ref.]	1.14 [1.03-1.26]	1.26 [1.15-1.38]	1.95 [1.75-2.18]	< 0.01
Multivariable aHR[1] + Modified CCI [95% CI]	1 [ref.]	1.18 [1.07-1.30]	1.35 [1.23-1.48]	2.08 [1.86-2.32]	< 0.01

Abbreviations: NAFLD, nonalcoholic fatty liver disease; NASH, nonalcoholic steatohepatitis; aHR, adjusted hazard ratio; CI, confidence interval; ref., referent
*The construction of the modified Charlson Comorbidity Index [CCI] is outlined in the Supplementary Methods.
[1]The multivariable-adjusted model accounted for the covariates outlined in the footnotes to Table 2, plus the modified Charlson Comorbidity Index [CCI]
[2]P for linear trend was estimated across NAFLD histology categories (modeled continuously), compared to simple steatosis; for details, see Methods.

Table S8. Array-Approach Sensitivity Analysis for an Unmeasured Confounder

<u>8A. Scenario #1</u>:

Confounder increases risk of all-cause mortality and is less prevalent in patients with NAFLD (the exposed).

1. We fixed the apparent relative risk (ARR) to the lower limit of the confidence interval of the pooled hazard ratio for all-cause mortality, comparing NAFLD patients to matched population comparators (adjusted hazard ratio, 1.93, 95% CI 1.86-2.00 [Table 2]).
2. We varied the association between the confounder and all-cause mortaity (RR_{CD}) from 1.00 to 4.00. We also varied the prevalence of the confounder from 10% to 50% in both the exposed group (P_{C1}) and the unexposed group (P_{C0}).

<u>Abbreviations used</u>:

ARR: Apparent (or observed) relative risk

RR_{CD}: Association between confounder and all-cause mortality

P_{C1}: Prevalence of confounder in the exposed group (NAFLD)

P_{C0}: Prevalence of the confounder in the unexposed group (population comparators)

$RR_{adjusted}$: Relative risk when adjusted for the unmeasured confounder

<u>Results:</u>

ARR	RR_{CD}	P_{C1}	P_{C0}	$RR_{adjusted}$	P_{C0}	$RR_{adjusted}$	P_{C0}	$RR_{adjusted}$	P_{C0}	$RR_{adjusted}$
1.86	1.0	0.3	0.1	1.9						
1.86	2.0	0.3	0.1	1.6						
1.86	3.0	0.3	0.1	1.4						
1.86	4.0	0.3	0.1	1.3						
1.86	4.5	0.3	0.1	1.3						
1.86	5.0	0.3	0.1	1.2						
1.86	1.0	0.4	0.1	1.9	0.2	1.9				
1.86	2.0	0.4	0.1	1.5	0.2	1.7				
1.86	3.0	0.4	0.1	1.3	0.2	1.5				
1.86	4.0	0.4	0.1	1.1	0.2	1.4				
1.86	4.5	0.4	0.1	1.1	0.2	1.4				
1.86	5.0	0.4	0.1	1.0	0.2	1.3				
1.86	1.0	0.5	0.1	1.9	0.2	1.9	0.3	1.9		
1.86	2.0	0.5	0.1	1.4	0.2	1.5	0.3	1.7		
1.86	3.0	0.5	0.1	1.2	0.2	1.4	0.3	1.5		
1.86	4.0	0.5	0.1	1.1	0.2	1.2	0.3	1.5		
1.86	4.5	0.5	0.1	1.0	0.2	1.2	0.3	1.4		
1.86	5.0	0.5	0.1	1.0	0.2	1.2	0.3	1.4		
1.86	1.0	0.6	0.1	1.9	0.2	1.9	0.3	1.9	0.4	1.9
1.86	2.0	0.6	0.1	1.3	0.2	1.4	0.3	1.6	0.4	1.7
1.86	3.0	0.6	0.1	1.1	0.2	1.2	0.3	1.5	0.4	1.6
1.86	4.0	0.6	0.1	1.0	0.2	1.1	0.3	1.3	0.4	1.5
1.86	4.5	0.6	0.1	0.8	0.2	1.1	0.3	1.3	0.4	1.5
1.86	5.0	0.6	0.1	0.8	0.2	1.0	0.3	1.2	0.4	1.5

<u>Explanation of Results</u>:

An unidentified confounding variable would have to have both (a) a relative risk for all-cause mortality of 4.5 or greater, and also (b) at least 50% difference in prevalence between groups (e.g. 60% prevalence among patients with NAFLD, compared to 10% in matched population comparators) to decrease the lower 95% confidence limit for the adjusted hazard ratio below 1.00.

8B. Scenario #2:

Confounder decreases risk of all-cause mortality, and is more prevalent among the exposed.

1. We fixed the observed relative risk (ARR) to the lower limit of the confidence interval of the hazard ratio for all-cause mortality, comparing NAFLD patients to population comparators (adjusted hazard ratio, 1.93, 95% CI 1.86-2.00 [Table 2]).
2. We varied the association between the confounder and incident HCC (RR_{CD}) from 1.00 to 4.00. We also varied the prevalence of the confounder from 10% to 50% in the NAFLD patients and matched population comparators.

Abbreviations used:

ARR: Apparent (or observed) relative risk

RR_{CD}: Association between confounder and all-cause mortality

P_{C1}: Prevalence of confounder in the exposed group

P_{C0}: Prevalence of the confounder in the unexposed group

$RR_{adjusted}$: Relative risk when adjusted for the unmeasured confounder

Results:

ARR	RR_{CD}	P_{C1}	P_{C0}	$RR_{adjusted}$	P_{C0}	$RR_{adjusted}$	P_{C0}	$RR_{adjusted}$	P_{C0}	$RR_{adjusted}$	P_{C0}	$RR_{adjusted}$
1.86	0.5	0.6	0.6	1.9								
1.86	0.4	0.6	0.6	1.9								
1.86	0.3	0.6	0.6	1.9								
1.86	0.2	0.6	0.6	1.9								
1.86	0.1	0.6	0.6	1.9								
1.86	0.5	0.5	0.6	1.7	0.5	1.9						
1.86	0.4	0.5	0.6	1.7	0.5	1.9						
1.86	0.3	0.5	0.6	1.7	0.5	1.9						
1.86	0.2	0.5	0.6	1.6	0.5	1.9						
1.86	0.1	0.5	0.6	1.6	0.5	1.9						
1.86	0.5	0.4	0.6	1.6	0.5	1.7	0.4	1.9				
1.86	0.4	0.4	0.6	1.6	0.5	1.7	0.4	1.9				
1.86	0.3	0.4	0.6	1.5	0.5	1.7	0.4	1.9				
1.86	0.2	0.4	0.6	1.4	0.5	1.6	0.4	1.9				
1.86	0.1	0.4	0.6	1.3	0.5	1.6	0.4	1.9				
1.86	0.5	0.3	0.6	1.5	0.5	1.6	0.4	1.8				
1.86	0.4	0.3	0.6	1.5	0.5	1.6	0.4	1.7				
1.86	0.3	0.3	0.6	1.4	0.5	1.5	0.4	1.7				
1.86	0.2	0.3	0.6	1.3	0.5	1.5	0.4	1.7				
1.86	0.1	0.3	0.6	1.2	0.5	1.4	0.4	1.6				
1.86	0.5	0.2	0.6	1.4	0.5	1.6	0.4	1.7	0.2	1.7		
1.86	0.4	0.2	0.6	1.4	0.5	1.5	0.4	1.6	0.2	1.7		
1.86	0.3	0.2	0.6	1.3	0.5	1.4	0.4	1.6	0.2	1.7		
1.86	0.2	0.2	0.6	1.2	0.5	1.3	0.4	1.5	0.2	1.7		
1.86	0.1	0.2	0.6	1.0	0.5	1.2	0.4	1.5	0.2	1.7		
1.86	0.5	0.1	0.6	1.4	0.5	1.5	0.4	1.6	0.2	1.6	0.1	1.9
1.86	0.4	0.1	0.6	1.3	0.5	1.4	0.4	1.5	0.2	1.6	0.1	1.9
1.86	0.3	0.1	0.6	1.2	0.5	1.3	0.4	1.4	0.2	1.5	0.1	1.9
1.86	0.2	0.1	0.6	1.1	0.5	1.2	0.4	1.4	0.2	1.8	0.1	1.9
1.86	0.1	0.1	0.6	0.9	0.5	1.1	0.5	1.1	0.2	1.7	0.1	1.9

Explanation of Results:

An unidentified confounder would need to have a relative risk for all-cause mortality of 0.1 or less and at least a 50% or greater difference in prevalence between groups (e.g. 10% among NAFLD patients, and 60% among population comparators), to decrease the lower 95% confidence limit for the adjusted hazard ratio below 1.00.

Supplementary References:

1. Svensk Förening för Patologi – Svensk Förening för Klinisk Cytologi V, April 4, 2019. http://www.svfp.se/foreningar/uploads/L15178/kvast/lever/Leverbiopsier2019.pdf; Accessed November 1, 2019.
2. Ludvigsson JF, Svedberg P, Olen O, et al. The longitudinal integrated database for health insurance and labour market studies (LISA) and its use in medical research. *Eur J Epidemiol* 2019;34(4):423-37. doi: 10.1007/s10654-019-00511-8 [published Online First: 2019/04/01]
3. Simon TG, Duberg AS, Aleman S, et al. Lipophilic Statins and Risk for Hepatocellular Carcinoma and Death in Patients With Chronic Viral Hepatitis: Results From a Nationwide Swedish Population. *Ann Intern Med* 2019 doi: 10.7326/M18-2753 [published Online First: 2019/08/20]
